# STOP Pain Project—Opioid Response in Pediatric Cancer Patients and Gene Polymorphisms of Cytokine Pathways

**DOI:** 10.3390/pharmaceutics14030619

**Published:** 2022-03-11

**Authors:** Giada Crescioli, Niccolò Lombardi, Laura Vagnoli, Alessandra Bettiol, Laura Giunti, Valentina Cetica, Maria Luisa Coniglio, Aldesia Provenzano, Sabrina Giglio, Roberto Bonaiuti, Alessandro Mugelli, Maurizio Aricò, Andrea Messeri, Alfredo Vannacci, Valentina Maggini

**Affiliations:** 1 Section of Pharmacology and Toxicology, Department of Neuroscience, Psychology, Drug Research and Children’s Health, University of Florence, 50139 Florence, Italy; giada.crescioli@unifi.it (G.C.); roberto.bonaiuti@unifi.it (R.B.); alessandro.mugelli@unifi.it (A.M.); alfredo.vannacci@unifi.it (A.V.); 2 Tuscan Regional Centre of Pharmacovigilance, 50122 Florence, Italy; 3 Pediatric Psychology Service, Meyer Children’s Hospital, 50139 Florence, Italy; laura.vagnoli@meyer.it; 4 Department of Experimental and Clinical Medicine, University of Florence, 50139 Florence, Italy; alessandra.bettiol@unifi.it; 5 Neuro-Oncology Unit, Department of Pediatric Oncology, Meyer Children’s Hospital, 50139 Florence, Italy; laura.giunti@meyer.it (L.G.); maurizio.arico@asl.pe.it (M.A.); 6 Laboratory of Neurogenetics, Pediatric Neurology Unit, Meyer Children’s Hospital, 50139 Florence, Italy; valentina.cetica@meyer.it; 7 Centre of Excellence, Division of Pediatric Oncology/Hematology, Meyer Children’s Hospital, 50139 Florence, Italy; marialuisa.coniglio@meyer.it; 8 Medical Genetics Unit, Department of Experimental and Clinical Biomedical Sciences “Mario Serio”, University of Florence, 50139 Florence, Italy; aldesia.provenzano@unifi.it (A.P.); sabrinar.giglio@unica.it (S.G.); 9 Unit of Medical Genetics, Department of Medical Sciences and Public Health, University of Cagliari, 09124 Cagliari, Italy; 10 Local Health Unit of Pescara—U.O.C. Pediatrics, 65124 Pescara, Italy; 11 Pain and Palliative Care Unit, Meyer Children’s Hospital, 50139 Florence, Italy; andrea.messeri@meyer.it; 12 Research and Innovation Center in Phytotherapy and Integrated Medicine (CERFIT), Referring Center for Phytotherapy, Careggi University Hospital, Tuscany Region, 50134 Florence, Italy

**Keywords:** opioid, pharmacogenetics, genetic polymorphisms, cancer, cytokines, pain, children

## Abstract

Moderate to severe cancer pain treatment in children is based on the use of weak and strong opioids. Pharmacogenetics play a central role in developing personalized pain therapies, as well as avoiding treatment failure and/or intolerable adverse drug reactions. This observational study aimed to investigate the association between IL-6, IL-8, and TNFα genetic single nucleotide polymorphisms (SNPs) and response to opioid therapy in a cohort of pediatric cancer patients. Pain intensity before treatment (PI_t0_) significantly differed according to IL-6 rs1800797 SNP, with a higher PI for A/G and G/G individuals (*p =* 0.017), who required a higher dose of opioids (*p =* 0.047). Moreover, compared to G/G subjects, heterozygous or homozygous individuals for the A allele of IL-6 rs1800797 SNP had a lower risk of having a PI_t0_ > 4. Dose_24h_ and Dose_tot_ were both higher in G/G individuals for TNFα rs1800629 (*p =* 0.010 and *p =* 0.031, respectively), while risk of having a PI_t0_ > 4 and a ∆_VAS_ > 2 was higher for G/G subjects for IL-6 rs1800795 SNP compared to carriers of the C allele. No statistically significant association between genotypes and safety outcomes was found. Thus, IL-6 and TNFα SNPs could be potential markers of baseline pain intensity and opioid dose requirements in pediatric cancer patients.

## 1. Introduction

In children under the age of 14, cancer incidence reaches 150 per million person-years in the populations of North America and Europe. The distribution of cancer type varies in age groups, with leukemia, central nervous system (CNS) tumors, and lymphomas as the most common types of cancers [1]. More recent evidence report ∼178 cases per million in Europe and North America, and up to ∼218 cases per million in West and Middle Africa [2].

According to 2012 World Health Organization guidelines [3], as for adults, moderate to severe cancer pain treatment in children is based on the use of weak and strong opioids. Inter-individual variability in response to these drugs has been observed both in adults and children [4], and currently, scientific research has focused its attention on clinical and genetic factors that can influence opioid treatment efficacy and safety [5]. Single nucleotide polymorphisms (SNPs) of genes involved in the pharmacokinetics and pharmacodynamics of drugs play a central role in developing personalized therapies for pain, avoiding failure of treatment or intolerable adverse drug reactions (ADRs) [6].

In cancer, progression of the disease, presence of metastasis, tissue infiltration, nerve damage, and the therapies themselves are all causes of pain [7]. Moreover, factors secreted from leukocytes in inflammatory infiltrates are hypothesized to operate as pain modulators both in inflamed tissues and in damaged peripheral nerves, leading to hyperalgesia and allodynia [8,9]. Furthermore, cytokines are associated with the alteration of the µ-opioid receptor level in neural and other immune cells and seem to be associated with peripheral sensitization [10,11]. Notably, cancer cells produce cytokines aberrantly both in hematological diseases and solid tumors [12], thus influencing pain expression and perception. Previous studies focused on adults have found some associations between interleukin-6 (IL-6), interleukin-8 (Il-8), tumor necrosis factor α (TNFα) SNPs, and clinical outcomes in cancer patients. 

IL-6, released in response to infection, trauma, and neoplasia, shows higher concentrations in various cancer types, and in particular in individuals who are carriers of both G alleles for its SNP rs1800795 [13]. IL-6 rs1800796 and rs1800797 SNPs are also associated with an over-expression of the IL-6 gene, particularly if considered as a haplotype with rs1800795 SNP [14]. The IL-6 haplotype has been associated with a higher duration of pain in patients with intervertebral disc disease and with more severe symptoms, including pain, in women affected by distal interphalangeal osteoarthritis [15]. The IL-8 rs4073 promoter SNP affects its concentrations and pain severity in patients with lung cancer and pancreatic adenocarcinoma [16,17]. TNFα is involved in pain facilitation and augmentation [18] and the presence of its rs1800629 SNP leads to an increased expression of TNFα both in vitro and in vivo [19]. Moreover, the same SNP is predictive of severe pain in lung cancer patients [20].

Particularly in children suffering from cancer, treating pain represents a challenge for clinicians. Among opioids, morphine is the most used active principle [21], but for some of these drugs, in particular oxycodone, tramadol, and methadone, clinicians must resort to off-label use [22,23], which may represent an additional risk in developing ADRs. 

Among genes associated with variability in response to opioid treatments, those involved in the cytokine pathway are still poorly investigated in children. Thus, the aim of this prospective observational cohort pilot study [24] was to investigate the association between IL-6 rs1800795, IL-6 rs1800796, IL-6 rs1800797, IL-8 rs4073, TNFα rs1800629 SNPs, and response to opioid therapy, both in terms of efficacy and safety, in a cohort of pediatric cancer patients. To the best of our knowledge, this is the first attempt to define predictive genetic factors of cytokine pathways modulating opioid profiles in oncologic pediatric patients.

## 2. Materials and Methods

The materials and methods of the Suitable Treatment for Oncologic Pediatric (STOP) Pain Study were described in 2018 [4]. Briefly, between June 2011 and April 2014, we prospectively enrolled all pediatric patients treated with opioids (morphine, codeine, oxycodone, fentanyl, and tramadol) for cancer pain, referring them to the Unit of Pain Therapy of Anna Meyer Children Hospital of Florence (Italy). The institutional review board at the Meyer Children’s Hospital approved the study. Informed consent was requested from participants’ parents. 

During the study period, we enrolled a total of 87 pediatric patients, but in this paper, we present results for patients with available genetic data for IL-6 rs1800795, rs1800796, rs1800797, IL-8 rs4073, and TNF-α rs1800629 SNPs (n = 74). We administered two structured questionnaires to enrolled children or to their parents in order to collect all patients’ demographic and clinical information (e.g., age, gender, allergies, medical history, concomitant illnesses, lifestyle, and body mass index, (BMI)), and demographic information on parents and the familial environment, respectively. We collected data regarding cancer diagnosis and the evolution of the disease from medical charts, while data concerning health conditions and parameters potentially predictive of treatment efficacy/safety were collected and considered for the analysis as confounding variables and/or effect modifiers. We recorded all data in anonymized, individual, coded case report forms (CRFs), and then we recorded all CRFs into an electronic database.

DNA analyses were performed on peripheral blood or mouth swab samples collected after patients’ recruitment [4]. Then, genetic, demographic, and clinical data were matched through patients’ reference codes. 

The dose of active principles other than morphine were converted into intravenous (IV) morphine equivalents (ME) according to the following calculations [25]: IV ME = oral oxycodone × 2/3 = IV tramadol × 10 = oral tramadol × 30 = oral codeine × 30 = IV fentanyl/100. When the direct conversion factor to IV ME was not available, the dosage was first converted to oral morphine equivalents and then to IV ME (3:1). The following three outcomes regarding dose were considered: (1) Dose_24h_ (mg/kg), the cumulative dose of IV ME administered during the first 24 h of treatment or titration phase; (2) Dose_tot_ (mg/kg): total dose of IV ME from Day 1 to the end of opioid treatment; (3) Dose_VAS=0_ (mg/kg): ME dose required to achieve total pain relief (visual analogic scale, VAS = 0).

Pain intensity (PI) was registered before the beginning of the treatment, again at every change of staff shift, and whenever patients showed pain using three different pain scales, according to the child’s age. The VAS was used for children older than 6 years of age. Wong & Baker FACES pain rating scale was administered to children 4–6 years old as an alternative outcome measure. The Face Legs Activity Cry Consolability (FLACC) scale was used for children who were unable to report the presence or the intensity of pain. The following three outcomes about pain intensity were considered: (1) Pi_t0_, pain intensity before treatment; (2) ∆_VAS_, difference between the pain intensity after 24 h of treatment and PI_to_; (3) Time_tot_ (hours), time needed to reach the lowest pain intensity reported by the patient.

We evaluated the occurrence of gastrointestinal (GI) side effects (nausea/vomiting, diarrhea, and constipation), central nervous system (CNS) effects (agitation, drowsiness, headache, and sedation), and the occurrence of any adverse effects (GI effects, CNS effects, and itching).

Categorical variables were presented as number and percentages and compared with Fisher’s exact test. Continuous variables were presented as mean and standard deviation (SD) and median and Interquartile Range (IQ Range). Means were compared with the Student’s *t*-test or by one-way ANOVA where appropriate, while differences for medians were checked using the Mann–Whitney test or the Kruskal–Wallis test. Logistic regression models were fitted to estimate the risk (odds ratio, (OR) and 95% Confidence Intervals, (CI)) of pain control and of side effects, and they were adjusted by age class, gender, Pi_t0_, diagnosis, and presence of metastasis. Statistical significance was considered for *p <* 0.05. Data management and statistical analysis were carried out using STATA 14.

## 3. Results

During the study period, we enrolled 87 subjects in the STOP Pain Project, but genetic analysis of IL-6 rs1800795, IL-6 rs1800796, IL-6 rs1800797, IL-8 rs4073, and TNFα rs1800629 SNPs were available for 74 patients. 

Table 1 shows the clinical and demographic characteristics of subjects included in the analysis compared to those subjects with missing genetic data (n = 13). Patients differed only for BMI (*p =* 0.048). Overall, 54.05% of the children were males, and most of them were aged more than 3 years (n = 53, 71.62%). The most frequently reported diagnoses were leukemia and lymphoma (41.89%), followed by sarcoma (18.92%) and osteosarcoma (17.57%). Only 21 patients reported the presence of metastasis. Half of the patients reported the oral cavity as the pain location, with a mean value of PI_t0_ of 4.32 points. Morphine was administered to 51 patients (68.92%), tramadol to 17 patients (22.97%), and only in three cases did we encounter the use of more than one active principle. In these three cases, patients were administered tramadol, codeine, fentanyl, and oxycodone. These medications were not administered simultaneously. Efficacy and safety parameters, overall and for subjects with missing genetic data, showed no differences.

The distribution of genotype frequencies agreed with the Hardy–Weinberg equilibrium (Table S1), and was consistent with 1000 Genome Project Data for the European population (EUR) [26]. 

Table 2 and Table 3 show clinical and demographic characteristics of subjects included in the present analysis according to their genotypes. PI_t0_ significantly differed according to IL-6 rs1800797 SNP, with a higher PI_t0_ for A/G and G/G individuals (*p =* 0.017).

Table 4 shows outcomes for opioid doses. Dose_tot_ was higher for individuals heterozygous and homozygous for the G allele of IL-6 rs1800797 SNP (*p =* 0.047). Dose_24h_ and Dose_tot_ were both higher in G/G individuals for TNFα rs1800629 (*p =* 0.010 and *p =* 0.031, respectively).

## 4. Discussion

Several pieces of evidence suggested a role of pro-inflammatory cytokines in the mediation of cancer-related symptoms, in particular pain. Pro-inflammatory cytokines released by activated glial cells cause hyperexcitability in pain transmission neurons. Consequently, presynaptic terminals release an exaggerated amount of substance-P and excitatory amino acids, producing a massive pain response [27]. In fact, nociceptor activity is modified and enhanced by cytokines released during inflammation processes or tissue damage, as in cancer [8]. The study of the influence of genetic variability on pain therapies, especially in children, is therefore essential.

In our study, we evaluated the role of several cytokine SNPs in opioid treatment efficacy/safety in a cohort of pediatric oncologic patients. In particular, we observed that subjects who were carriers of the A allele for the IL-6 rs1800797 SNP had a lower mean value of PI before starting opioid treatments and needed a lower total amount of the considered medications, compared to carriers of the G allele. Similarly, patients homozygous for the G allele of IL-6 rs1800795 SNP had a higher PI before they were administered with opioids and a higher risk of worsening in pain evaluation. In fact, a value of ∆_VAS_ > 2 showed that their PI after 24 h of treatment was higher than their PI at baseline. Moreover, individuals who were homozygous for the G allele of TNFα rs1800629 SNP required a higher opioid dose, both during the first 24 h of treatment and during the entire analgesic treatment.

IL-6 gene is localized to chromosome 7p21. The IL-6 rs1800795 SNP affects transcription, altering serum levels of IL-6 with the G allele, which is associated with higher concentrations of IL-6 in plasma [14]. As mentioned above, IL-6 rs1800797 and IL-6 rs1800795 SNPs are considered as haplotypes and are associated with an over-expression of IL-6 gene [14]. TNFα has been implicated in various diseases, and genetic SNPs in the promoter region, such as rs1800629, could modulate protein expression [19].

IL-6 and TNFα pathways suggest a strict connection between inflammation mediators, which may stimulate pre-malignant cell proliferation, angiogenesis, and metastasis, and, tumor cells, which reversely stimulate other cells to produce pro-inflammatory and pro-angiogenic factors [28]. In our sample, the most reported diagnoses were leukemia and lymphomas, sarcoma and osteosarcomas, and brain tumors. High serum levels of IL-6 have also been observed in these cancer conditions. In particular, compared to healthy children and adolescents, pediatric patients with acute myeloid leukemia showed higher concentrations of IL-6 and TNFα [29]. Up-regulation of cytokines in the bone microenvironment may contribute to peripheral and central components of cancer-induced bone pain, as osteoclasts are derived from monocyte/macrophage hematopoietic lineage and express receptors for TNFα and IL-6 [30]. Moreover, IL-6, IL-8, and TNFα resulted in being overexpressed in the serum of adult and pediatric patients affected by brain tumors [28,31]. Thus, our results concerning PI and, consequently opioid dose, could be related both to the effects of genetic SNPs and to the type of cancer experienced by our patients, underlining the influence of both cancer and genetic characteristics on baseline PI and the efficacy of opioid treatments. Evidence of a between-system adaptation, particularly between the opioid receptors system and pro-inflammatory cytokines, may explain to some extent the need for a higher amount of morphine and the occurrence of tolerance. Neuroinflammation and the release of proinflammatory cytokines are the key molecular mechanisms for the above-mentioned between-system adaptation. In fact, TNFα, IL-18, IL-1β, and IL-6 in microglia and astrocytes are associated with alteration of the µ-opioid receptor level in neural and other immune cells [10]. Interestingly, cytokines seem to be associated with peripheral sensitization, making nociceptors overly responsive to previously subthreshold levels of peripheral stimulus. Following the development of central sensitization, nociceptive neurons potently signal to the CNS, resulting in hyperalgesia and allodynia. Given the evidence that supports pro-inflammatory cytokine action in the occurrence of opioid-induced hyperalgesia, new therapeutic approaches that aim to decrease pro-inflammatory factors or increase anti-inflammatory cytokines may guarantee a novel tool in therapy improvement of cancer-related pain [11]. The identification of new potential targets for the development of novel pain therapeutics may overcome the limitations encountered so far, improving patients’ outcomes.

Our results are in line with those already published in the literature for adult cancer patients. IL-6 serum levels were positively related to the severity of a score, including, in adult patients with gastrointestinal cancer, the six most severe cancer-related symptoms (i.e., fatigue and pain) [32]. Although we did not observe an association between PI outcomes and TNFα rs1800629 SNP, the higher dose of opioids administered to these patients (Dose_24h_ and Dose_tot_) could be considered as a proxy of their PI. The ability to quantify pain within a pediatric population is not simple. In fact, even if all scales used referred to a 0-to-10-point range, the use of different scales for pain evaluation may have affected these results in terms of homogeneity. An additive model for TNF rs1800629 SNP was predictive of severe pain in adults with lung cancer [20], and in a study by Reyes-Gibby et al. [33], G/G lung cancer patients required a higher daily dose of ME. The same study reported higher pain values for individual homozygous for the G allele of IL-6 rs1800795 SNP.

Specific evidence for IL-6 rs1800797 and cancer-related pain is not available in literature. A study on adult patients suffering from sciatica demonstrated that the number of days with back and leg pain and days on sick leave were significantly higher among subjects with the IL6 rs1800797, rs1800796, rs1800795, and rs13306435 haplotypes [15]. Another study from Kämäräinen and colleagues showed that the G allele for IL-6 rs1800797 and rs1800795 SNPs were more common in women with distal interphalangeal osteoarthritis, and that the risk of symptomatic distal interphalangeal osteoarthritis was increased in carriers of a haplotype with the G allele in all three promoter SNPs of IL-6 (rs1800797, rs1800795, and rs1800796) [34].

IL-8 rs4073 SNP has been significantly associated with severe pain among adult patients with pancreatic adenocarcinoma [17]. On the contrary, results for adults with lung cancer are controversial. Two studies reported IL-8 rs4073 SNP to be associated with severe pain [16,35], while Hsiu-Ling and colleagues did not find the same associative trend with symptoms distress (including fatigue, sleep disturbance, depression, pain, and cognitive problems) [36]. Probably, as was the case for the article published by Hsiu-Ling and colleagues, due to the small number of patients enrolled, our study was unable to find this association.

Overall, results obtained in our study could be primarily related to the well-known existing differences between adults and children: developmental pharmacokinetic [37], off-label use [3], and medication characteristics. These variables could have influenced the relationship between the SNPs studied and the outcomes for opioid doses. Moreover, focusing on treatment efficacy, the use of equianalgesic tables raises concerns. The possible inaccuracy in the calculation of ME could be related to the use of different equivalence ranges within or between equianalgesic tables, especially for formulations containing an opioid and other analgesics [38].

The present study has some limitations. First, we could not establish whether any concomitant non-opioid analgesics used in our sample may have affected PI evaluation. Since some agents may synergize with opioids and/or have similar genetic influences, having this information would be very important. Another limitation was represented by the mixed nature of pain in cancer patients. In fact, pediatric cancer-related pain can vary from acute, procedure-related pain to breakthrough pain, and considering these specific mechanisms, it is essential to manage pain effectively. Thus, it was difficult to evaluate the influence of type and location of the primary cancer or the presence of metastases on PI outcomes.

On the contrary, our evidence underlines the significance of pharmacogenetic studies. Remarkably, this is the first study attempting to evaluate the role of cytokine pathway gene SNPs in pediatric oncology and their impact on opioid efficacy and safety. Although we did not observe any significant association between genetic factors and opioid-related ADRs, our data showed that patients’ genetic profiles could be proxies for baseline PI and related treatment requirements.

In this context, only future studies with larger sample sizes could determine the prompt impact of these SNPs on opioid efficacy for cancer-related pain treatment in children.

## 5. Conclusions

Our results underline that IL-6 and TNFα SNPs could be potential markers of baseline pain intensity and opioid dose requirements in pediatric cancer patients. Knowledge of them could therefore address opioid treatment management in pediatric oncology. Further studies evaluating new strategies to modulate interactions between cytokines and the opioid system may identify novel therapeutic approaches in this subset in clinical practice.

## Figures and Tables

**Table 1:** Characteristics of STOP Pain patients and efficacy and safety parameters overall and for subjects with missing genetic data.

	Overall	Missing	
	n (%)	n (%)	*p*-Value
	74	13	
Gender			
Male	40 (54.05)	9 (69.23)	0.375 *
Female	34 (45.95)	4 (30.77)	
Age (months)			
0–36	21 (28.38)	2 (15.38)	
>36–144	29 (39.19)	6 (46.15)	0.695 *
>144	24 (32.43)	5 (38.46)	
BMI (percentile)			
<25th	24 (32.43)	5 (38.46)	
25th–75th	14 (18.92)	5 (38.46)	0.048 *
≥75th	23 (31.08)	-	
Missing	13 (17.57)	3 (23.08)	
Diagnosis			
Brain Tumor	4 (5.41)	2 (15.38)	
Histiocytosis	4 (5.41)	-	
Leukemia and Lymphoma	31 (41.89)	3 (23.08)	
Neuroblastoma	6 (8.11)	-	0.387 *
Osteosarcoma	13 (17.57)	4 (30.77)	
Sarcoma	14 (18.92)	4 (30.77)	
Other	2 (2.70)	-	
Metastasis			
No	53 (71.26)	11 (84.62)	0.500 *
Yes	21 (28.38)	2 (15.38)	
Pain location			
Abdominal	11 (14.86)	1 (7.69)	
Oral cavity	37 (50.00)	6 (46.15)	0.521 *
Skeletal–Muscle	10 (13.51)	4 (30.77)	
Other	16 (21.62)	2 (15.38)	
Pain Intensity			
PI_to_			
Mean ± SD	4.32 ± 2.146	4.46 ± 2.436	0.835 ^§^
Median (IQ Range)	4 (3–6)	4 (4–6)	0.696 ^#^
∆_VAS_			
Mean ± SD	−2.36 ± 2.303	−1.69 ± 3.881	0.389 ^§^
Median (IQ Range)	−2 (−4–−1)	−1 (−4–0)	0.525 ^#^
Time _tot_ (hours)			
Mean ± SD	139.59 ± 65.539	145.19 ± 55.632	0.773 ^§^
Median (IQ Range)	133.75 (100–192)	128.00 (96–195)	0.766 ^#^
Pain Intensity PI_to_ grouped			
≤4	40 (54.05)	7 (53.85)	1.000 *
>4	34 (45.95)	6 (46.15)	
Drug			
Morphine	51 (68.92)	9 (69.23)	
Tramadol	17 (22.97)	2 (15.38)	
Oxycodone	1 (1.35)	1 (7.69)	0.483 *
Codeine	2 (2.70)	-	
more than one	3 (4.05)	1 (7.69)	
Dose (mg/kg)			
Dose_24h_^a^			
Mean ± SD	0.38 ± 0.209	0.39 ± 0.266	0.875 ^§^
Median (IQ Range)	0.41 (0.193–0.492)	0.44 (0.167–0.498)	0.966 ^#^
Dose_tot_			
Mean ± SD	2.50 ± 1.823	2.96 ± 2.052	0.401 ^§^
Median (IQ Range)	2.17 (1.200–3.309)	2.83 (1.330–3.933)	0.439 ^#^
Dose_VAS=0_^b^			
Mean ± SD	0.73 ± 1.029	0.89 ± 1.121	0.639 ^§^
Median (IQ Range)	0.32 (0.095–0.906)	0.45 (0.132–1.048)	0.438 ^#^
Side effects			
Gastrointestinal			0.738 *
Yes	19 (25.68)	4 (30.77)	
No	55 (74.32)	9 (69.23)	
CNS			0.641 *
Yes	8 (10.81)	2 (15.38)	
No	66 (89.19)	11 (84.62)	
Total			0.753 *
Yes	24 (32.43)	5 (38.46)	
No	50 (67.57)	8 (66.67)	

^a^ Dose_24h_ was available for 72 patients. ^b^ Dose_VAS=0_ was available for 69 patients with genetic analysis and 11 patients with missing genetic data. * Fisher’s exact test. ^§^ Student’s *t*-test. ^#^ Mann–Whitney test.

**Table 2:** Characteristics of the 74 subjects included in the STOP Pain Project and efficacy and safety parameters according to IL6 G174C, G572C, and A597G polymorphisms.

	IL-6 rs1800795			*p*-Value	IL-6 rs1800796		*p*-Value	IL-6 rs1800797			*p*-Value
	G/G	G/C	CC		G/G	G/C		A/A	A/G	G/G	
	n (%)	n (%)	n (%)		n (%)	n (%)		n (%)	n (%)	n (%)	
	35	30	9		63	11		8	29	37	
Gender											
Male	17 (48.57)	18 (60.00)	5 (55.56)	0.683 *	36 (57.14)	4 (36.36)	0.326 *	6 (75.00)	16 (55.17)	18 (48.65)	0.399 *
Female	18 (51.43)	12 (40.00)	4 (44.44)		27 (42.86)	7 (63.64)		2 (25.00)	13 (44.83)	19 (51.35)	
Age (months)											
0–36	14 (40.00)	5 (16.67)	2 (22.22)	0.156 *	18 (28.57)	3 (27.27)	1.000 *	2 (25.00)	6 (20.69)	13 (35.14)	0.542 *
>36–144	9 (25.71)	15 (50.00)	5 (55.56)		25 (39.68)	4 (36.36)		4 (50.00)	14 (48.28)	11 (29.73)	
>144	12 (34.29)	10 (33.33)	2 (22.22)		20 (31.75)	4 (36.36)		2 (25.00)	9 (31.03)	13 (35.14)	
BMI (percentile)											
<25th	11 (31.43)	11 (36.67)	2 (22.22)	0.752 *	21 (33.33)	3 (27.27)	0.886 *	1 (12.50)	11 (37.93)	12 (32.43)	0.607 *
25th–75th	7 (20.00)	4 (13.33)	3 (33.33)		11 (17.46)	3 (27.7)		3 (37.50)	3 (10.34)	8 (21.62)	
≥75th	9 (25.71)	11 (36.67)	3 (33.33)		20 (31.75)	3 (27.27)		3 (37.50)	10 (34.48)	10 (27.03)	
Missing	8 (22.86)	4 (13.33)	1 (11.11)		11 (17.46)	2 (18.18)		1 (12.50)	5 (17.24)	7 (18.92)	
Diagnosis											
Brain Tumor	4 (11.43)	-	-	0.299 *	2 (3.17)	2 (18.18)	0.433 *	-	-	4 (10.81)	0.171 *
Histiocytosis	3 (8.57)	1 (3.33)	-		4 (6.35)	-		-	1 (3.45)	3 (8.11)	
Leukemia and Lymphoma	10 (28.57)	15 (50.00)	6 (66.67)		25 (39.68)	6 (54.55)		6 (75.00)	15 (51.72)	10 (27.03)	
Neuroblastoma	5 (14.29)	1 (3.33)	-		6 (9.52)	-		-	2 (6.90)	4 (10.81)	
Osteosarcoma	6 (17.14)	5 (16.67)	2 (22.22)		11 (17.46)	2 (18.18)		2 (25.00)	3 (10.34)	8 (21.62)	
Sarcoma	7 (20.00)	6 (20.00)	1 (11.11)		13 (20.63)	1 (9.09)		-	6 (20.69)	8 (21.62)	
Other	-	2 (6.67)	-		2 (3.17)	-		-	2 (6.90)	-	
Metastasis											
No	24 (68.57)	22 (73.33)	7 (77.78)	0.878 *	45 (71.43)	8 (72.73)	1.000 *	7 (87.50)	23 (79.31)	23 (62.16)	0.222 *
Yes	11 (31.43)	8 (26.67)	2 (22.22)		18 (28.57)	3 (27.27)		1 (12.50)	6 (20.69)	14 (37.84)	
Pain location											
Abdominal	7 (20.00)	3 (10.00)	1 (11.11)	0.834 *	9 (14.29)	2 (18.18)	0.728 *	1 (12.50)	2 (6.90)	8 (21.62)	0.537 *
Oral cavity	17 (48.57)	16 (53.33)	4 (44.44)		30 (47.62)	7 (63.64)		5 (62.50)	15 (51.72)	17 (45.95)	
Skeletal–Muscle	3 (8.57)	5 (16.67)	2 (22.22)		9 (14.29)	1 (9.09)		1 (12.50)	6 (20.69)	3 (8.11)	
Other	8 (22.86)	6 (20.00)	2 (22.22)		15 (23.81)	1 (9.09)		1 (12.50)	6 (20.69)	9 (24.32)	
Pain Intensity PI_t0_											
Mean ± SD Median (IQ Range)	4.86 ± 2.315 5 (3–7)	3.97 ± 1.810 4 (3–5)	3.44 ± 2.186 3 (2–5)	0.104 ** 0.223 ^†^	4.40 ± 2.189 4 (3–6)	3.91 ± 1.921 4 (3–5)	0.490 ** 0.434 ^†^	3.12 ± 2.100 3 (1.5–5)	3.79 ± 1.800 4 (3–5)	5 ± 2.224 5 (3–7)	0.017 ** 0.040 ^†^
Pain Intensity PIto Grouped											
≤4	17 (48.57)	18 (60.00)	5 (55.56)	0.683 *	32 (50.79)	8 (72.73)	0.208 *	5 (62.50)	19 (65.52)	16 (43.24)	0.196 *
>4	18 (51.43)	12 (40.00)	4 (44.44)		31 (49.21)	3 (27.27)		3 (37.50)	10 (34.48)	21 (56.76)	
Drug											
Morphine	25 (71.43)	21 (70.00)	5 (55.56)	0.610 *	42 (66.67)	9 (81.82)	0.387 *	4 (50.00)	20 (68.97)	27 (72.97)	0.452 *
Tramadol	7 (20.00)	6 (20.00)	4 (44.44)		16 (25.40)	1 (9.09)		4 (50.00)	6 (20.69)	7 (18.92)	
Oxycodone	1 (2.86)	-	-		1 (1.59)	-		-	-	1 (2.70)	
Codeine	-	2 (6.67)	-		1 (1.59)	1 (9.09)		-	2 (6.90)	-	
more than one	2 (5.71)	1 (3.33)	-		3 (4.76)	-		-	1 (3.45)	2 (5.41)	

* Fisher’s exact test. ^†^ Kruskal–Wallis test. ** ANOVA.

**Table 3:** Characteristics of the 74 subjects included in the STOP Pain Project and efficacy and safety parameters according to IL 8 T251A and TNFα G308A polymorphisms.

	IL-8 rs4073			*p*-Value	TNFα rs1800629		*p*-Value
	T/T	T/A	A/A		G/G	G/A	
	n (%)	n (%)	n (%)		n (%)	n (%)	
	24	30	20		63	11	
Gender							
Male	15 (62.50)	15 (50.00)	10 (50.00)	0.641 *	31 (49.21)	9 (81.82)	0.055 *
Female	9 (37.50)	15 (50.00)	10 (50.00)		32 (50.79)	2 (18.18)	
Age (months)							
0–36	6 (25.00)	9 (30.00)	6 (30.00)	0.985 *	18 (28.57)	3 (27.27)	1.000 *
>36–144	10 (41.67)	12 (40.00)	7 (35.00)		25 (39.68)	4 (36.36)	
>144	8 (33.33)	9 (30.00)	7 (35.00)		20 (31.75)	4 (36.36)	
BMI (percentile)							
<25th	7 (29.17)	8 (26.67)	9 (45.00)	0.770 *	20 (31.75)	4 (36.36)	0.840 *
25th–75th	6 (25.00)	5 (16.67)	3 (15.00)		11 (17.46)	3 (27.27)	
≥75th	6 (25.00)	11 (36.67)	6 (30.00)		20 (31.75)	3 (27.27)	
Missing	5 (20.83)	6 (20.00)	2 (10.00)		12 (19.05)	1 (9.09)	
Diagnosis							
Brain Tumor	2 (8.33)	2 (6.67)	-	0.502 *	4 (6.35)	-	0.536 *
Histiocytosis	1 (4.17)	2 (6.67)	1 (5.00)		3 (4.76)	1 (9.09)	
Leukemia and Lymphoma	12 (50.00)	14 (46.67)	5 (25.00)		26 (41.27)	5 (45.45)	
Neuroblastoma	3 (12.50)	1 (3.33)	2 (10.00)		6 (9.52)	-	
Osteosarcoma	3 (12.50)	5 (16.67)	5 (25.00)		9 (14.29)	4 (36.36)	
Sarcoma	2 (8.33)	6 (20.00)	6 (30.00)		13 (20.63)	1 (9.09)	
Others	1 (4.17)	-	1 (5.00)		2 (3.17)	-	
Metastasis							
No	16 (66.67)	23 (76.67)	14 (70.00)	0.722 *	45 (71.43)	8 (72.73)	1.000 *
Yes	8 (33.33)	7 (23.33)	6 (30.00)		18 (28.57)	3 (27.27)	
Pain location							
Abdominal	4 (20.00)	5 (16.67)	4 (20.00)	0.554 *	8 (12.70)	3 (27.27)	0.504 *
Oral cavity	11 (55.00)	11 (36.67)	11 (55.00)		33 (52.38)	4 (36.36)	
Skeletal–Muscle	2 (10.00)	5 (16.67)	2 (10.00)		8 (12.70)	2 (18.18)	
Other	3 (15.00)	9 (30.00)	3 (15.00)		14 (22.22)	2 (18.18)	
Pain Intensity PI_t0_							
Mean ± SD Median (IQ Range)	4.04 ± 2.476 3.5 (2–6)	4.33 ± 2.218 4 (3–5)	4.65 ± 1.598 4.5 (3–6)	0.651 ** 0.614 ^†^	4.38 ± 2.136 4 (3–6)	4.00 ± 2.280 3 (3–5)	0.558 ** 0.590 ^†^
Pain Intensity PIt_0_ Grouped							
≤4	14 (58.33)	16 (53.33)	10 (50.00)	0.876 *	34 (53.97)	6 (54.55)	1.000 *
>4	10 (41.67)	14 (46.67)	10 (50.00)		29 (46.03)	5 (45.45)	
Drug							
Morphine	16 (66.67)	19 (63.33)	16 (80.00)	0.610 *	46 (73.02)	5 (45.45)	0.086 *
Tramadol	7 (29.17)	6 (20.00)	4 (20.00)		13 (20.63)	4 (36.36)	
Oxycodone	-	1 (3.33)	-		-	1 (9.09)	
Codeine	1 (4.17)	1 (3.33)	-		2 (3.17)	-	
more than one	-	3 (10.00)	-		1 (9.09)	1 (9.09)	

* Fisher’s exact test. ** ANOVA. ^†^ Kruskal-Wallis test.

**Table 4:** Association between genetic factors and opioid dose outcomes.

	Dose_24h_ (mg/kg) Mean ± SD Median (IQ Range)	*p*-Value	Dose_tot_ (mg/kg) Mean ± SD Median (IQ Range)	*p*-Value	Dose_VAS=0_ (mg/kg) Mean ± SD Madian (IQ Range)	*p*-Value
IL-6-rs1800795						
Co-dominant						
C/C	0.30 ± 0.140 0.25 (0.192–0.456)	0.482 ** 0.623 ^†^	1.42 ± 0.793 1.12 (0.882–2.239)	0.156 ** 0.166 ^†^	0.69 ± 0.741 0.48 (0.092–1.034)	0.951 ** 0.986 ^†^
G/C	0.37 ± 0.214 0.42 (0.194–0.504)		2.73 ± 2.041 2.18 (1.260–4.471)		0.78 ± 1.168 0.323 (0.100–1.157)	
G/G	0.40 ± 0.219 0.42 (0.200–0.547)		2.58 ± 1.752 2.28 (1.217–3.378)		0.70 ± 1.000 0.24 (0.093–0.905)	
Dominant						
C/C	0.30 ± 0.140 0.25 (0.192–0.456)	0.265 ** 0.340 ^†^	1.41 ± 0.793 1.12 (0.417–2.239)	0.056 ** 0.058 ^†^	0.69 ± 0.741 0.48 (0.092–1.034)	0.890 ** 0.908 ^†^
G/C + G/G	0.39 ± 0.215 0.42 (0.194–0.547)		2.65 ± 1.877 2.185 (1.260–3.495)		0.74 ± 1.070 0.28 (0.097–0.905)	
Recessive						
C/C + G/C	0.36 ± 0.200 0.40 (0.192–0.480)	0.403 ** 0.600 ^†^	2.42 ± 1.904 2.16 (1.058–3.309)	0.708 ** 0.471 ^†^	0.76 ± 1.068 0.324 (0.097–1.096)	0.830 ** 0.947 ^†^
G/G	0.40 ± 0.219 0.42 (0.200–0.547)		2.58 ± 1.752 2.28 (1.217–3.378)		0.70 ± 1.000 0.24 (0.093–0.905)	
IL-6 rs1800796						
Co-dominant						
G/C	0.37 ± 0.193 0.40 (0.255–0.570)	0.987 ** 0.925 ^†^	2.95 ± 1.806 2.89 (1.430–4.378)	0.380 ** 0.298 ^†^	0.49 ± 0.695 0.22 (0.100–0.655)	0.423 ** 0.512 ^†^
G/G	0.38 ± 0.213 0.42 (0.192–0.480)		2.42 ± 1.829 2.06 (1.125–3.276)		0.77 ± 1.074 0.33 (0.093–1.034)	
IL-6 rs1800797						
Co-dominant						
G/G	0.41 ± 0211 0.43 (0.225–0.527)	0.253 ** 0.323 ^†^	2.62 ± 1.725 2.37 (1.430–3.378)	0.164 ** 0.130 ^†^	0.74 ± 1.002 0.29 (0.093–0.906)	0.689 ** 0.691 ^†^
A/G	0.37 ± 0.217 0.41 (0.205–0.542)		2.66 ± 2.066 2.18 (1.222–4.471)		0.81 ± 1.164 0.33 (0.100–1.157)	
A/A	0.27 ± 0.137 0.22 (0.162–0.401)		1.34 ± 0.741 1.32 (0.703–1.950)		0.45 ± 0.638 0.25 (0.051–0.517)	
Dominant						
A/A	0.27 ± 0.137 0.22 (0.162–0.401)	0.134 ** 0.157 ^†^	1.34 ± 0.741 1.32 (0.703–1.950)	0.057 ** 0.047 ^†^	0.45 ± 0.638 0.25 (0.051–0.517)	0.412 ** 0.394 ^†^
G/G + A/G	0.39 ± 0.213 0.422 (0.208–0.527)		2.64 ± 1.868 2.23 (1.222–3.495)		0.77 ± 1.068 0.33 (0.100–1.005)	
Recessive						
G/G	0.41 ± 0211 0.43 (0.225–0.527)	0.231 ** 0.330 ^†^	2.62 ± 1.725 2.37 (1.430–3.378)	0.558 ** 0.297 ^†^	0.74 ± 1.002 0.29 (0.093–0.906)	0.970 ** 0.688 ^†^
A/G + A/A	0.35 ± 0.204 0.37 (0.184–0.480)		2.37 ± 1.931 1.87 (1.058–3.276)		0.73 ± 1.069 0.32 (0.095–1.034)	
IL-8 rs 4073						
Co-dominant						
A/A	0.42 ± 0.185 0.46 (0.252–0.0570)	0.576 ** 0.383 ^†^	2.58 ± 1.912 2.59 (1.137–3.942)	0.807 ** 0.886 ^†^	0.75 ± 1.122 0.32 (0.121–0.905)	0.689 ** 0.975 ^†^
A/T	0.36 ± 0.217 0.42 (0.145–0.480)		2.33 ± 1.69 2.05 (1.200–2.965)		0.83 ± 1.20 0.39 (0.076–1.005)	
T/T	0.36 ± 0.217 0.37 (0.196–0.468)		2.64 ± 1.960 2.14 (1.277–3.684)		0.57 ± 0.598 0.26 (0.130–0.966)	
Dominant						
A/A	0.42 ± 0.185 0.46 (0.252–0.0570)	0.292 ** 0.172 ^†^	2.58 ± 1.912 2.59 (1.137–3.942)	0.812 ** 0.752 ^†^	0.75 ± 1.122 0.32 (0.121–0.905)	0.913 ** 0.825 ^†^
A/T + T/T	0.36 ± 0.215 0.40 (0.149–0.480)		2.47 ± 1.806 2.09 (1.200–3.276)		0.72 ± 1.003 0.31 (0.920–1.005)	
Recessive						
A/A + A/T	0.38 ± 0.206 0.44 (0.193–0.505)	0.640 ** 0.441 ^†^	2.43 ± 1.770 2.21 (1.200–3.201)	0.649 ** 0.835 ^†^	0.80 ± 1.159 0.33 (0.092–0.906)	0.040 ** 0.947 ^†^
T/T	0.36 ± 0.217 0.37 (0.196–0.468)		2.64 ± 1.960 2.14 (1.277–3.684)		0.57 ± 0.598 0.26 (0.130–0.966)	
TNF-α rs1800629						
Co-dominant						
A/G	0.23 ± 0.136 0.19 (0.119–0.330)	0.010 ** 0.012 ^†^	1.40 ± 1.196 1.06 (0.535–1.826)	0.031 ** 0.023 ^†^	0.26 ± 0.241 0.18 (0.092–0.482)	0.120 ** 0.162 ^†^
G/G	0.40 ± 0.209 0.43 (0.250–0.547)		2.69 ± 1.85 2.28 (1.260–3.495)		0.81 ± 1.090 0.33 (0.095–1.105)	

** ANOVA, analysis adjusted for: age class, gender, Pi_t0_, diagnosis, metastasis. ^†^ Kruskal–Wallis test. Associations between genetic factors and PI outcomes, before and after opioid treatment, are reported in Table 5. Risk of having a PI_t0_ > 4 and a ∆_VAS_ > 2 was higher for G/G subjects for IL-6 rs1800795 SNP compared to carriers of the C allele, both heterozygous or homozygous: OR 5.00 (95% CI, 1.105–22.651) and OR 4.75 (95% CI, 1.271–17.785), respectively. Compared to G/G subjects, heterozygous or homozygous individuals for the A allele of IL-6 rs1800797 SNP had a lower risk of having a PI_t0_ > 4, OR 0.14 (95% CI, 0.030–0.621). Logistic regression did not show any statistically significant association between genotypes and safety outcomes following opioid treatments (Table S2).

**Table 5:** Association between genetic factors and pain intensity (PI) outcomes before and after opioid treatment.

	PI_t0_		Δ_VAS_		Responders		Time_tot_	
	n >4/≤4	OR (95% CI)	n >2/≤2	OR (95% CI)	n No/Yes	OR (95% CI)	Mean ± SD Median (IQ Range)	*p*-Value
IL-6 rs1800795								
Co-dominant								
C/C	4/5	1	5/4	1	0/9	1	107.22 ± 49.246 118.00 (96.00–133.00)	0.290 ** 0.235 ^†^
G/C	12/18	0.51 (0.075–3.497)	15/15	1.19 (0.232–6.145)	10/20	0.61 (0.149–2.485)	144.45 ± 68.914 148.50 (78.00–201.00)	
G/G	18/17	2.97 (0.356–24.804)	6/29	5.44 (0.881–33.646)	9/26	-	143.76 ± 65.459 138.00 (102.00–191.50)	
Dominant								
C/C	4/5	1	5/4	1	0/9	1	107.22 ± 49.246 118.00 (96.00–133.00)	0.114 ** 0.092 ^†^
G/C + G/G	30/35	0.97 (0.161–5.855)	21/44	2.15 (0.447–10.312)	19/46	-	144.08 ± 66.546 144.00 (100.00–192.00)	
Recessive								
C/C + G/C	16/23	1	20/19	1	10/29	1	135.86 ± 66.237 133.00 (78.00–192.00)	0.608 ** 0.697 ^†^
G/G	18/17	5.00 (1.105–22.651)	6/29	4.75 (1.271–17.785)	9/26	0.95 (0.250–3.604)	143.76 ± 65.459 138.00 (102.00–191.50)	
IL-6 rs1800796								
Co-dominant								
G/C	3/8	1	4/7	1	3/8	1	152.23 ± 69.778 150.00 (100.00–192.00)	0.492 ** 0.584 ^†^
G/G	31/32	3.73 (0.502–27.708)	22/41	0.65 (0.145–2.944)	16/47	0.90 (0.165–4.969)	137.39 ± 65.106 133.00 (99.00–192.00)	
IL-6 rs1800797								
Co-dominant								
A/A	3/5	1	4/4	1	1/7	1	141.78 ± 64.620 133.00 (102.00–185.50)	0.648 ** 0.660 ^†^
A/G	10/19	0.35 (0.043–2.854)	16/13	0.59 (0.105–3.380)	8/21	0.19 (0.167–2.185)	142.48 ± 68.160 147.00 (96.00–192.00)	
G/G	21/16	3.30 (0.665–17.255)	6/31	3.75 (0.586–24.049)	10/27	0.22 (0.020–2.550)	119.00 ± 64.73 119.00 (68.50–163.25)	
Dominant								
A/A	3/5	1	22/44	1	18/48	1	142.09 ± 65.682 141.00 (100.00–192.00)	0.350 ** 0.365 ^†^
G/G + A/G	31/35	1.06 (0.161–7.016)	4/4	0.78 (0.150–4.009)	1/7	4.84 (0.475–49.223)	119.00 ± 64.73 119.00 (68.50–163.25)	
Recessive								
G/G	21/16	1	6/31	1	10/27	1	141.78 ± 64.620 133.00 (102.00–185.50)	0.776 ** 0.833 ^†^
A/G + A/A	13/24	0.14 (0.030–0.621)	20/17	0.18 (0.048–0.668)	9/28	1.27 (0.322–5.018)	137.40 ± 67.263 134.50 (96.00–192.00)	
IL-8 rs 4073								
Co-dominant								
A/A	10/10	1	6/14	1	4/16	1	135.55 ± 83.109 128.50 (74.50–199.00)	0.770 ** 0.832 ^†^
A/T	14/16	1.31 (0.291–5.924)	9/21	1.08 (0.273–4.263)	8/22	0.73 (0.150–3.529)	135.85 ± 56.42 136.25 (100.00–189.50)	
T/T	10/14	1.00 (0.213–4.719)	11/13	0.48 (0.111–2.043)	7/17	0.55 (0.101–2.972)	147.65 ± 61.751 147.00 (101.50–191.75)	
Dominant								
A/A	10/10	1	6/14	1	4/16	1	135.55 ± 83.109 128.50 (74.50–199.00)	0.749 ** 0.794 ^†^
A/T + T/T	24/30	1.16 (0.300–4.472)	20/34	0.77 (0.221–2.700)	15/39	0.65 (0.149–2.834)	141.02 ± 58.580 141.00 (100.00–191.50)	
Recessive								
A/A + A/T	24/26	1	15/35	1	12/38	1	135.73 ± 67.545 133.00 (99.00–192.00)	0.468 ** 0.544 ^†^
T/T	10/14	0.85 (0.239–3.041)	11/13	0.45 (0.143–1.436)	7/17	0.67 (0.179–2.538)	147.65 ± 61.751 147.00 (101.50–191.75)	
TNFα rs1800629								
Co-dominant								
A/G	5/6	1	3/8	1	4/7	1	119.54 ± 87.222 118.00 (59.00–130.00)	0.274 ** 0.095 ^†^
G/G	29/34	0.93 (0.151–5.696)	23/40	0.45 (0.091–2.244)	15/48	1.32 (0.282–6.142)	143.09 ± 61.21 144.00 (101.00–192.00)	

** ANOVA, analysis adjusted for: age class, gender, Pi_t0_, diagnosis, metastasis. ^†^ Kruskal–Wallis test.

## Data Availability

The data presented in this study are available in the article, in the Supplementary Materials, and from the corresponding author upon reasonable request.

## Supplementary Materials

The following supporting information can be downloaded at: https://www.mdpi.com/article/10.3390/pharmaceutics14030619/s1: Table S1: Distribution of the SNPs in eight genes in the studied population; Table S2: Association between genetic factors and safety outcomes after opioid treatment.
