# Supplementary material for: STOP Pain Project—Opioid Response in Pediatric Cancer Patients and Gene Polymorphisms of Cytokine Pathways
Source: Pharmaceutics. 2022 Mar 11;14(3):619. doi: 10.3390/pharmaceutics14030619 (PMC8953705; doi:10.3390/pharmaceutics14030619)
Supplement: Supplementary file 1 [file pharmaceutics-14-00619-s001.zip › pharmaceutics-1624175-supplementary.pdf]

pharmaceutics

MDPI

Article

STOP Pain Project—Opioid Response in Pediatric Cancer Patients and Gene Polymorphisms of Cytokine Pathways

Giada Crescioli [1,2,†], Niccolò Lombardi [1,2,*,†], Laura Vagnoli [3], Alessandra Bettiol [4], Laura Giunti [5], Valentina Cetica [6], Maria Luisa Coniglio [7], Aldesia Provenzano [8], Sabrina Giglio [8,9], Roberto Bonaiuti [1], Alessandro Mugelli [1], Maurizio Aricò [5,10], Andrea Messeri [11], Alfredo Vannacci [1,2,†] and Valentina Maggini [1,12,*,†]

Table S1. Distribution of the SNPs in eight genes in the studied population.

Gene	N *	%	Hardy-Weinberg (*p*-Value)	MAF °
**IL6 rs1800795**				
GG	35	47.30	0.720	0.416
GC	30	40.54		
CC	9	12.16		
**IL-6 rs1800796**				
GG	63	85.13	0.930	0.048
GC	11	14.86		
CC	0	0		
**IL-6 rs1800797**				
AA	8	10.81	0.950	0.408
AG	29	39.19		
GG	37	50.00		
**IL8 rs4073**				
TT	24	32.43	0.950	0.421
TA	30	40.54		
AA	20	27.03		
**TNFα rs1800629**				
GG	63	85.13	0.930	0.134
GA	11	14.86		
AA	0	0		

* All patients have Caucasian origins. ° European Population of 1000 Genomes Project data (dbSNP-1000Genome-EUR).

Table S2. Association between genetic factors and safety outcomes after opioid treatment.

	ADRs		Gastrointestinal		SNC	
	**N** Yes/No	**OR** (95% CI)	**N** Yes/No	**OR** (95% CI)	**N** Yes/No	**OR** (95% CI)
**IL-6 rs1800795**						
Co-dominant						
C/C	3/6	1	2/7	1	1/8	1

G/C	9/21	0.76 (0.134–4.282)	8/22	1.21 (0.181–8.106)	3/27	0.18 (0.004–8.050)
G/G	12/23	0.53 (0.083–3.377)	9/26	1.03 (0.137–7.678)	4/31	0.01 (0.00–1.439)
Dominant						
C/C	3/6	1	2/7	1	1/8	1
G/C+G/G	21/44	0.66 (0.125–3.430)	17/48	1.13 (0.183–7.042)	7/58	0.07 (0.001–3.604)
Recessive						
C/C+G/C	12/27	1	10/29	1	4/35	1
G/G	12/23	0.66 (0.180–2.387)	9/26	1.29 (0.396–4.253)	4/31	0.03 (0.00–1.716)
**IL-6 rs1800796**						
Co-dominant						
G/C	3/8	1	2/9	1	1/10	1
G/G	21/42	1.58 (0.303–8.225)	17/46	2.10 (0.332–13.249)	7/56	2.23 (0.099–49.893)
**IL-6 rs1800797**						
Co-dominant						
A/A	3/5	1	2/6	1	1/7	1
A/G	9/20	0.64 (0.102–4.028)	7/22	0.85 (0.112–6.521)	4/25	0.24 (0.006–8.909)
G/G	12/25	0.42 (0.01–2.879)	10/27	0.87 (0.108–7.065)	3/34	0.01 (0.00–1.074)
Dominant						
A/A	3/5	1	2/6	1	1/7	1
G/G+ A/G	21/45	0.09 (0.02–3.584)	17/49	0.86 (0.125–5.979)	7/59	0.09 (0.002–3.584)
Recessive						
G/G	12/25	1	10/27	1	3/34	1
A/G+ A/A	12/25	1.68 (0.466–6.084)	9/28	1.01 (0.295–3.860)	5/32	35.93 (0.990–1304.652)
**IL-8- rs4073**						
Co-dominant						
A/A	8/12	1	7/13	1	1/19	1
A/T	13/17	1.38 (0.374–5.117)	12/18	1.30 (0.333–5.082)	4/26	NA
T/T	3/21	NA	0/24	-	3/21	NA
Dominant						
A/A	8/12	1	7/13	1	1/19	1
A/T+T/T	16/38	0.74	12/42	0.54	7/47	NA

		(0.220–2.465)		(0.152–1.940)		
Recessive						
A/A+A/T	21/29	1	19/31	1	5/45	1
T/T	3/21	0.18 (0.038–0.819)	0/24	1.19 (0.319–4.422)	3/21	4.24 (0.412–43.539)
**TNFα rs1800629**						
Co-dominant						
A/G	4/7	1	3/8	1	1/10	1
G/G	20/43	0.81 (0.174–3.738)	16/47	1.07 (0.202–5.621)	7/56	0.68 (0.049–9.402)

Analysis adjusted for: age class, gender, Pi_{t0}, diagnosis, metastasis. NA: not assessable.
